# A large and feasible national survey representative of population exposure to outdoor gamma radiation in urban areas

**DOI:** 10.3389/fpubh.2024.1388783

**Published:** 2024-05-30

**Authors:** Carmela Carpentieri, Andrea Maiorana, Marco Ampollini, Sara Antignani, Mario Caprio, Vinicio Carelli, Carlo Cordedda, Christian Di Carlo, Francesco Bochicchio

**Affiliations:** ^1^Italian National Institute of Health / National Center for Radiation Protection and Computational Physics, Rome, Italy; ^2^Telecom Italia SpA, Rome, Italy

**Keywords:** environmental radioactivity, gamma radiation, outdoor radiation exposure, national survey, urban environments

## Abstract

**Background:**

Although data on outdoor gamma radiation are available for many countries, they have generally been obtained with measurements performed in undisturbed environments instead of in urban areas where most of the population lives. Only one large national survey, with on-site measurements in urban areas, has been identified worldwide, probably due to high costs (e.g., personnel and instrumentation) and difficulties in selecting measuring points.

**Methods:**

A campaign of outdoor gamma radiation measurements has been carried out in the entire Italian territory. All measurement points were selected at the infrastructures of an Italian telecommunications company as representatives of all the possible situations of outdoor exposure to gamma radiation for population in urban areas. Ten replicates of portable gamma (X) detectors carried out all the measurements.

**Results:**

Approximately 4,000 measurements have been performed. They are distributed across 2,901 Italian municipalities, accounting for 75% of the Italian population. The national population-weighted mean of the gamma ambient dose equivalent rate (ADER) is 117 nSv h^−1^, and it ranges from 62 to 208 nSv h^−1^ and from 40 to 227 nSv h^−1^ for 21 regions and 107 provinces, respectively. The average variability at the municipal level, in terms of the coefficient of variation (CV) is 21%, ranging from 3 to 84%. The impact of land coverage and the distance from a building on the outdoor gamma radiation level was assessed with complementary measurements, leading to differences ranging from −40 to 50% and to 50%, respectively.

**Conclusion:**

A representative campaign of outdoor gamma dose rate measurements has been performed in Italy, only in urban areas, to assess the exposure effect due to outdoor gamma radiation on the population. It is the largest national campaign in urban areas worldwide, with a total of 3,876 on-site measurements. The land coverage and the distance from surrounding buildings were recognized to strongly affect outdoor gamma radiation levels, leading to high variability within small areas. The collaboration with a company that owns a network of facilities on a national territory as dense as the residing population made this survey feasible and affordable. Other countries might adopt this methodology to conduct national surveys in urban environments.

## 1 Introduction

Gamma radiation has been classified as a Group 1 carcinogen to humans (1), meaning that “sufficient evidence” for carcinogenic effects has been proven by epidemiological studies on humans. Moreover, no threshold has been proven to exist that could consider an increase in cancer risk due to exposure to ionizing radiation as negligible (2, 3).

People suffer external exposure mainly due to gamma rays of terrestrial origin in Earth’s crust, released by the decay of naturally occurring radionuclides (NOR) mostly belonging to K-40, U-238, and Th-232 decay chains (usually referred to as “primordial radionuclides”).

The United Nations Scientific Committee on the Effects of Atomic Radiation (UNSCEAR) reported a worldwide population-weighted average of the absorbed dose rate in air of 58 nGy h–1, ranging from 45 to 140 nGy h-1, due to gamma rays of terrestrial origin, based on measurements in 45 countries around the world in undisturbed environments, i.e., open fields disturbed as little as possible by human activities (4).

Measurements in urban environments are more suitable for assessing the actual exposure of population to outdoor gamma radiation since most people live in cities. As for undisturbed environments, outdoor gamma radiation level in built-up areas is mainly contributed by the gamma rays originating from natural radionuclides in the upper soil layer (4), i.e., up to a maximum depth of 50 cm (5), the activity concentration of which is mainly controlled by that in the underlying geological basement (6). At the same time, urbanization structures, i.e., buildings and road pavements, affect outdoor gamma radiation levels in urban areas (7, 8). In the case of building materials and land coverages characterized by high NOR activity concentrations, an increase in outdoor gamma radiation dose rate values ranging from 4 to 50% [e.g., 9, 10] was reported (11, 12), especially in scenarios with high building density (13, 14) or approaching the building perimeter (10). In contrast, when made of materials containing low NOR activity concentrations, land coverage attenuates terrestrial gamma rays and reduces the outdoor gamma dose rate (12, 15).

Most of the data on outdoor gamma radiation levels published in the literature refer to undisturbed environments since they were obtained mainly from national monitoring networks for radiological emergencies [e.g., (16, 17)], which are typically placed outside cities or by solving Beck’s equation (4) with natural radionuclide activity concentrations in soils as the input (18).

In studies focused on assessing exposure due to outdoor gamma radiation in populations, the measurements were typically conducted by car-borne or air-borne surveys over portions of the country, including areas both inside and outside cities [e.g., (7, 19–21)].

At the local level, some studies collected measurements of outdoor gamma radiation in urban environments within limited areas (8, 22–27), while, at the national level, the UNSCEAR reported that less than 10% of national surveys on outdoor gamma radiation exposure have been conducted in urban environments (4). This significantly low percentage is justified by considering the difficulties associated with the selection of measurement points, assuring high population and territory coverage as well as easy access, the need for personnel to carry out measurements at locations that are typically distant from each other, the high costs for instrumentation, and the long duration of the study. All these factors lead to only three national surveys, of which only one has a high number of measurements, as found in literature, based on on-site measurements in urban environments – i.e., Clouvas, Xanthos (28), Clouvas, Xanthos (29) in Greece with 1,039 measurements, Svoukis and Tsertos (30) in Cyprus with 70 measurements, and Zerquera, Alonso (31) in Cuba with 142 measurements.

Only a few results have been published about outdoor gamma radiation measurements in urban areas in Italy: few examples in limited portions of Italian territory are available (12, 32, 33). The only national survey carried out in Italy dates back to 1972, and it included 1,365 on-site outdoor gamma radiation measurements distributed both in urban environments and outside inhabited centers, covering 19 of the 21 Italian regions (34).

The present study collected 3,876 on-site outdoor gamma radiation measurements in urban areas, nearly three times those of the previous national survey, covering all 21 Italian regions, all 107 Italian provinces, and 2,901 Italian municipalities (approximately 40% of the total), which accounts for approximately 75% of the Italian population. The present study was conducted only in urban areas, where most Italian population lives. Consequently, it assesses a more reliable estimate of the actual exposure of the Italian population to outdoor gamma radiation. This survey is currently the most extensive collection of on-site outdoor gamma radiation measurements within urban areas at the national level found in the literature.

Urban areas are typically characterized by different building materials used for constructing roads and buildings and different installation conditions (e.g., thickness), leading to an increase in the spatial variability of outdoor gamma radiation levels within built-up environments compared to undisturbed ones (14). The latter is affected by spatial variability typically ranging from 2 to 10%, considering the same geological baseline (12, 35), primarily due to the inhomogeneities of the NOR activity concentration in soil (36, 37). In contrast, some authors reported a variability ranging from 14 to 56% in terms of the coefficient of variation (CV) on outdoor gamma radiation measurements for nearly 300 cities worldwide (13). The availability of more than four measurements for 74 Italian municipalities within the same urban area enabled the estimation of the range of spatial variability of outdoor gamma radiation levels in urban areas. Furthermore, a small complementary campaign of measurements in a limited and controlled area was carried out to quantify the impact of the distance from a building made of materials with high natural radionuclide activity concentrations and using different land coverages on the outdoor gamma radiation level.

The methodology adopted, based on a collaboration with a national telecommunications company for measurement point selection, is feasible and affordable. It leads to high population and territory coverage, allowing the overcoming of many difficulties associated with national surveys in urban areas. Other countries might adopt it to carry out such a measurement campaign.

## 2 Methods

### 2.1 Sampling strategy

The outdoor gamma dose rate measurements have been included in a national radon survey to measure the radon activity concentration in a large sample of Italian telecommunication company workplaces (38). The measurement points were chosen at the national telecommunication company telephone exchanges—i.e., buildings housing plant equipment and telephone switches and buildings with offices and small underground inspection rooms commonly used for telecommunications network management (Table 1). These infrastructures are located almost entirely in urban areas, with few exceptions that were excluded *a priori*.

**Table 1:** Distribution of measurement points over Italian telecommunication company workplaces.

Measurement point typology	Number
Inspection rooms	1,895
Telephone Exchanges	1,936
Buildings	45
Total	3,876

All these facilities belong to a network covering the national territory as densely as the resident population, which is strictly connected to a number of telephone lines. This connection made it possible to avoid the phase of selecting measurement points in urban areas on the national territory to guarantee high population coverage, as the measurement points were already available with addresses. Furthermore, access to all measurement points was simple with a single authorization from the company, unlike what would have happened in the case of points chosen *a priori* on a map. In total, 3,876 infrastructures in urban areas in all the Italian territory have been chosen as measurement points, leading to the most extensive collection of on-site outdoor gamma radiation measurements within urban areas at the national level worldwide.

Moreover, an agreement with the company made their employees, communication channels, and measurement instrumentation available, leading to a streamlined organization in a short time and economy.

### 2.2 Sample description and representativeness

The Italian administrative situation considered in this study refers to 2023: The Italian territory is divided into 7,901 municipalities, 107 provinces, and 21 regions (19 regions and 2 autonomous provinces). Municipalities, the smallest territorial unit, are administratively aggregated to form provinces, which are aggregated into regions. The population of the municipalities, provinces, and regions is surveyed by the Italian National Institute of Statistics (ISTAT) on the latest National Census available (39).

The distribution of municipalities surveyed is not homogeneous among the various regions (see Table 2), reflecting the density of the infrastructure network connected to the residing population. The telecommunications company’s network is less dense in areas with fewer inhabitants, and the same facilities serve more municipalities. In contrast, more inspection rooms, telephone exchanges, and office buildings are concentrated in highly inhabited and urbanized areas, with more measurement points available.

**Table 2:** Present study coverage of Italian municipalities and population by region (the administrative situation of Italy, regions, and municipalities, refers to 2023).

Italian region	Total measurements	Municipalities with measurements in the region	Total municipalities in the region	Percentage of regional municipalities coverage	Percentage of regional population coverage
Abruzzo	130	99	305	32%	78%
Basilicata	102	81	131	62%	79%
Calabria	179	149	404	37%	67%
Campania	473	313	550	57%	80%
Emilia-Romagna	256	167	330	51%	80%
Friuli Venezia Giulia	109	84	215	39%	70%
Lazio	159	74	378	20%	79%
Liguria	51	27	234	12%	54%
Lombardia	643	570	1,504	38%	71%
Marche	119	78	225	35%	72%
Molise	33	30	136	22%	65%
Piemonte	276	230	1,180	19%	69%
Puglia	254	202	257	79%	91%
Sardegna	109	92	377	24%	57%
Sicilia	331	242	391	62%	84%
Toscana	121	93	273	34%	66%
Umbria	83	43	92	47%	82%
Valle d’Aosta	18	18	74	24%	50%
Veneto	230	170	563	30%	56%
Aut. Prov. Bolzano	100	73	104	70%	79%
Aut. Prov. Trento	86	51	58	88%	95%
**Italy**	**3,876**	**2,901**	**7,901**	**37%**	**74%**

At the national level, the municipality coverage—i.e., the total surveyed municipalities of the Italian ones—is 37%, corresponding to a population coverage of 74% (Table 2). When the regional scale is considered, the municipality coverage is, on average, 42%—obtained as the mean of the municipality coverages of the 21 Italian regions—ranging from 12 to 88%; the population coverage is, on average, 73%—obtained as the mean of the population coverages of the 21 Italian regions—ranging from 50 to 95% (Table 2). Even in regions with a low percentage of municipality coverage, the population coverage is higher than 50% (Table 2).

All Italian regions were surveyed: Puglia, Sicilia, and Basilicata have the highest percentage of municipalities involved in the study; in contrast, Lazio, Piemonte, and Liguria have the lowest percentage of municipalities. Indeed, for Lazio and Piemonte, most of the measurements were carried out in cities with a high population, Rome and Torino, respectively.

At a provincial scale, in terms of municipalities, the coverage is, on average, 43% with a first quartile of 24% and a third quartile of 60%, while in terms of population, the coverage is, on average, 70% with a first quartile of 60% and a third quartile of 84%. The complete table of the data is provided as Supplementary material. At the national level, 37% of the Italian municipalities have been surveyed, corresponding to 74% of the Italian population (Table 2).

According to the protocol used (described in the “Measuring instruments and protocol” section), the measurement points were not chosen *a priori* relative to the distance from the nearest building and the type of road coverage. Therefore, the measurement points represent all the possible situations of outdoor exposure to gamma radiation by the population in urban areas.

At regional and national levels, as well as for most Italian provinces, the sample selected is representative of Italian population’s exposure to outdoor gamma radiation in urban areas, considering the high number of measurements, the high population coverage, and the absence of selection bias due to the distribution of the telecommunication network on the territory. The Italian territory and population coverage—at national, regional, and provincial scales—is much higher than that in the previous Italian national survey conducted in 1972 (see §3).

### 2.3 Measuring instruments and protocol

The measurements of gamma dose rates were carried out by ten replicates of the Automess 6,150 AD-b/H portable X-gamma detector already provided by the radioprotection laboratory of the national telecommunications company. The detector consists of a cylindrical organic scintillator with a diameter of 3 in, height of 3 in, and density of 1.032 g cm^−3^. The overall instrument dimensions are 353 × 195 × 96 mm^**3**^. The range of the instrument measurement energy is from 20 keV to 7 MeV, and the instrumental background is approximately 1 nSv h^−1^.

All the gamma detectors employed were calibrated simultaneously with a 333 kBq ± 10% calibration Cs–137 sealed source certified by the Physikalisch-Technische Bundesanstalt (PTB). The instrument’s output operational quantity is the ambient dose equivalent, H* (12). The instrumental efficiency for a standard outdoor spectrum of gamma radiation from natural sources is about the same as that of Cs-137 (662 keV) (40).

The measurements were performed by the employees of the telecommunications company, following an agreed protocol. The instrument, held in hand at nearly 1 m from the ground (41) and positioned in any direction, was kept in measure for 1 min, and then, another 1-min measurement was performed by rotating the instrument at 180° to the previous configuration, but keeping the same position at the same height. Subsequently, the 2-min gamma dose rate average returned by the instrument was reported (42). To ensure the stability of the gamma detector calibration for quality control, the team conducted an average dose rate measurement (for 2 min) at a fixed point (e.g., their office) before starting outdoor measurements every day. The measurement point was chosen at the center of the closest place for pedestrians to pass, nearest to the external entrance of the telephone exchanges or buildings, and 10 m from the maintenance hole covering the underground inspection room. No instructions were given about the distance from surrounding buildings and land coverage: this made the measurement points representative of all the possible outdoor gamma exposure situations in urban areas.

### 2.4 Data analysis

In total, 3,876 outdoor ambient dose equivalent rate measurements were collected on the Italian territory. The measurements within the same municipality were averaged, and 2,901 values of municipality-specific outdoor gamma radiation dose rate were obtained. Then, the municipality data were averaged on a provincial basis using the municipality population on the total provincial population as weight. A mean population-weighted outdoor gamma radiation dose rate value was obtained for each of the 107 Italian provinces. The same scheme was applied for regions, and 21 mean population-weighted outdoor gamma radiation dose rate values were obtained.

To evaluate the variability of outdoor gamma radiation within urban environments, data obtained from 74 Italian municipalities have been used. In these municipalities, more than four measurements were collected within the same urban area, and the coefficient of variation (CV) was computed. In each of these municipalities, the CV has been calculated as the ratio between the standard deviation of the measurements of the municipality and their average.

## 3 Results

**Figure 1:**
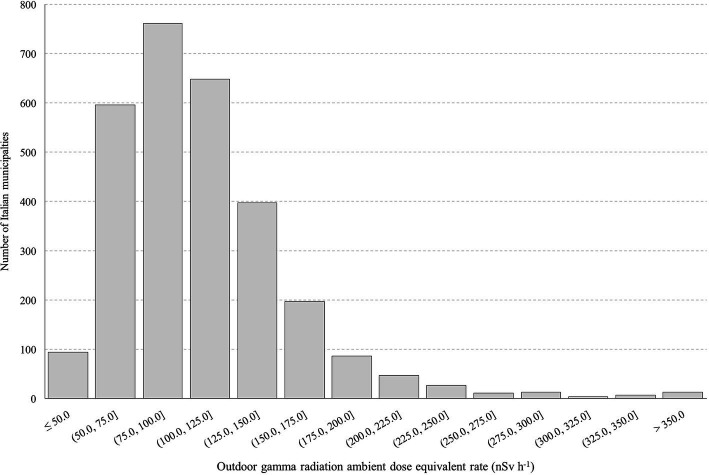
Histogram that collects the mean outdoor ambient dose equivalent rate values of 2,901 Italian municipalities.

The statistical distribution of the outdoor gamma radiation results in the 2,901 Italian municipalities is reported in Figure 1. Table 3 reports the 21 mean population-weighted outdoor gamma radiation dose rate values. For each region, the maximum and minimum municipality values are also reported in Table 3. The resulting national mean gamma population-weighted ambient dose equivalent is 117 nSv h^−1^, ranging from 62 to 208 nSv h^−1^ among regions.

**Table 3:** Population-weighted average outdoor gamma dose rate per regions.

Italian Region	Population-weighted mean	Population-weighted standard deviation	Maximum municipal value	Minimum municipal value
	(nSv h^−1^)	(nSv h^−1^)	(nSv h^−1^)	(nSv h^−1^)
Abruzzo	72	7	148	48
Basilicata	94	9	218	60
Calabria	137	8	218	55
Campania	190	42	492	12
Emilia-Romagna	93	17	187	48
Friuli Venezia Giulia	66	2	159	47
Lazio	208	36	377	54
Liguria	70	15	120	35
Lombardia	103	16	340	10
Marche	62	10	285	42
Molise	74	3	148	51
Piemonte	134	12	297	60
Puglia	87	12	217	50
Sardegna	136	10	242	78
Sicilia	95	30	497	20
Toscana	75	10	169	35
Umbria	66	5	148	40
Valle d’Aosta	168	15	230	100
Veneto	89	18	167	42
Aut. Prov. Bolzano	188	9	492	12
Aut. Prov. Trento	139	8	497	61
**Italy**	**117**	**45**		

The Italian regions with the highest outdoor gamma dose rate values are Lazio and Campania—208 and 190 nSv h^−1^, respectively.

**Figure 2:**
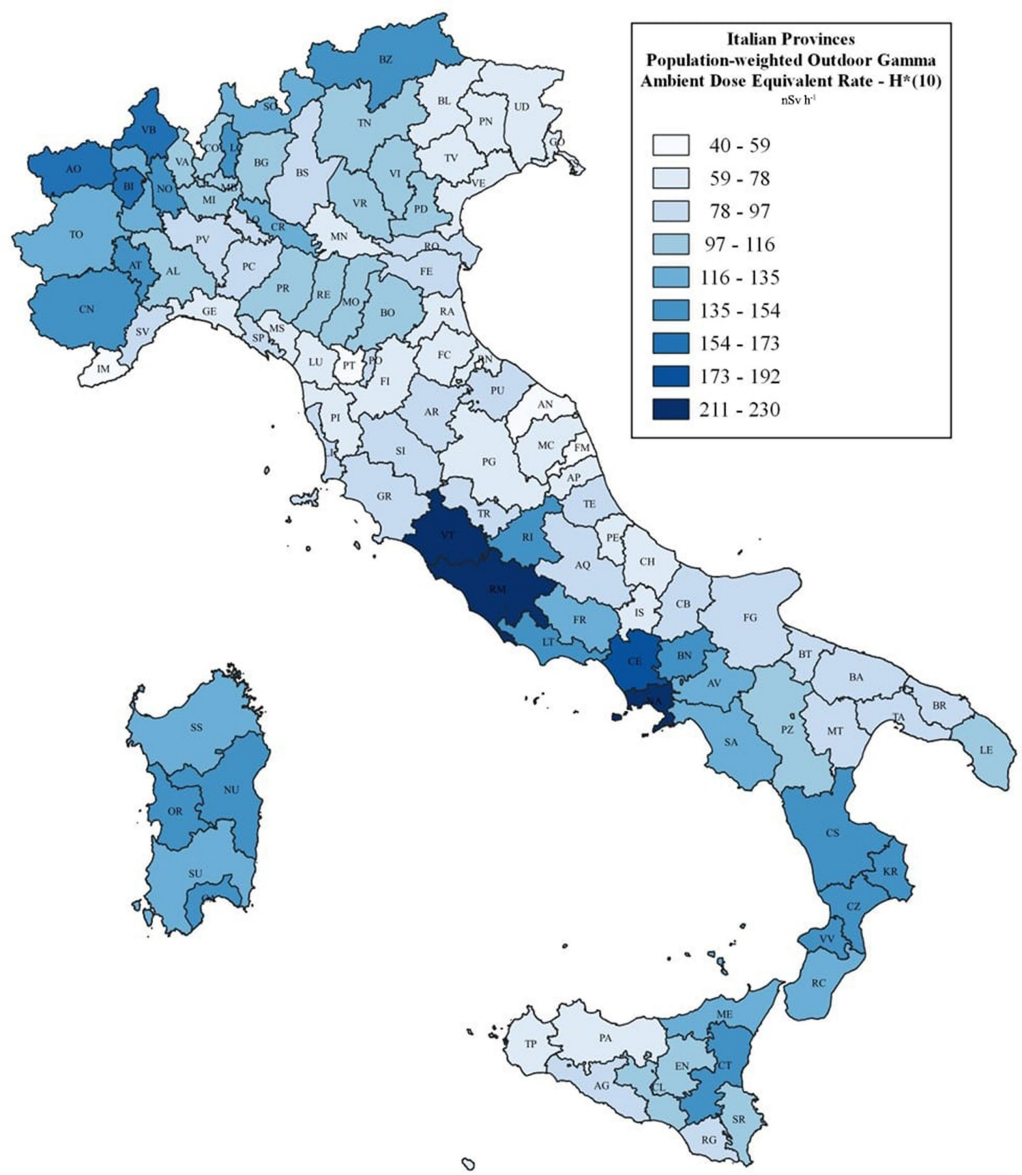
Weighted mean outdoor gamma dose rate averages by province on a graduated scale.

Figure 2 shows the population-weighted mean of the provincial gamma dose rate value on a graduated scale, ranging from 40 to 227 nSv h^−1^. The complete table of the data at the provincial scale is provided as the Supplementary material, reporting all the mean population-weighted values of the outdoor gamma radiation dose rate, as well as the maximum and minimum values in each province.

**Figure 3:**
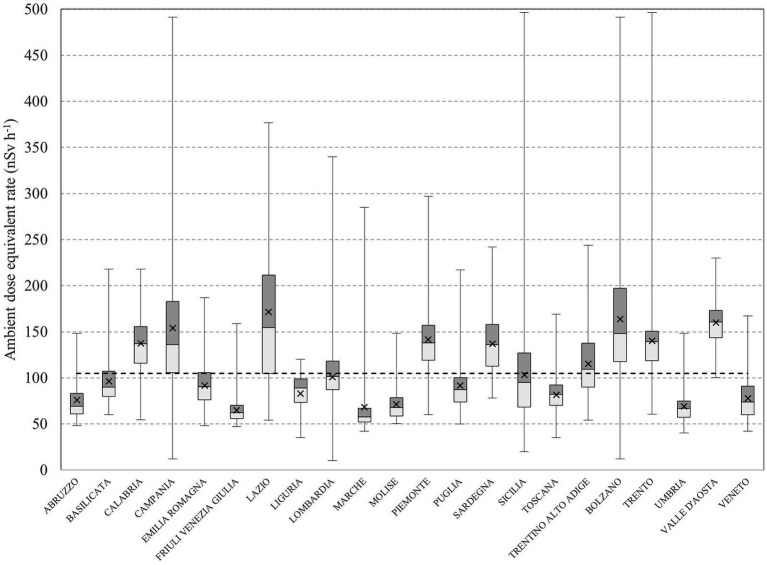
Box plot with regional data on the outdoor gamma dose rate. The dotted line is the Italian national arithmetic mean, i.e., not population-weighted.

In Figure 3, the boxplot with the regional data is shown, reporting the regional arithmetic mean (cross), the first quartile (box lower), the third quartile (box upper), and the median of outdoor gamma radiation measurements. Moreover, the national arithmetic mean, i.e., not weighted, was plotted (dot line), along with regional whisker plots, showing variations in measured outdoor gamma dose rates in regional municipalities. For most Italian regions, the data are characterized by a skewed distribution, with a long tail toward high values (see Discussion section).

The resulting mean annual effective dose for the Italian population living in urban areas is 0.13 mSv y–1, ranging from 0.07 to 0.22 mSv y^−1^. These values were obtained by assuming a mean outdoor occupancy factor of 0.2 (i.e., about 1,800 h y^−1^) and a conversion factor of 0.61 (43) from the ambient dose equivalent to the effective dose. The conversion factor was obtained under the assumptions of isotropic geometry exposure (13) and the mean energy of natural gamma radiation was approximately 1 MeV (44).

## 4 Discussion

**Figure 4:**
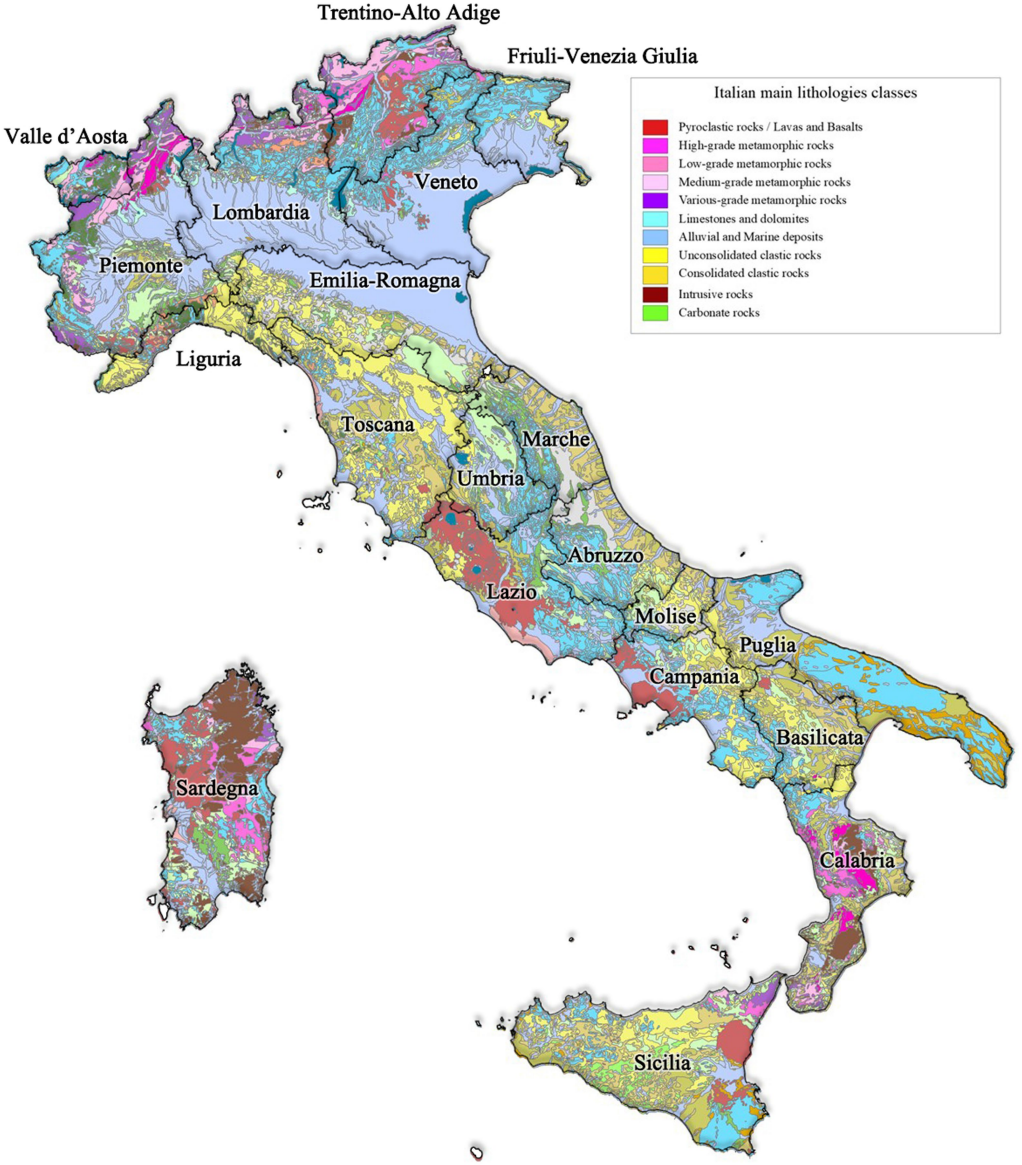
Geo-lithological map of Italy, only lithologies of interest were reported in the legend. The map was rearranged from the geo-lithological map of Italy released by the Italian Institute for Environmental Protection and Research.

The activity concentration of natural radionuclides in the upper soil layer, mostly controlled by geological baseline characteristics, was recognized as the main influencing factor for outdoor gamma radiation. The areas affected by volcanic activity, past or present, show the highest outdoor gamma dose rate values since the geological baseline is made of magmatic rocks, referred to as “pyroclastic rocks, lavas, and basalts” or “intrusive rocks” in the Italian geo-lithological map reported in Figure 4, characterized by high NOR activity concentrations (45–47), e.g., Lazio and Campania, Eastern Sicily (48), Southern Calabria, Sardegna, as well as Northern Piemonte (49–51). The Alps area in Northern Italy is also characterized by the presence of magmatic products of old volcanic bodies, referred to as “metamorphic rocks” in Figure 4, with high potassium-40 activity concentrations (51–53). Consequently, it is also associated with high outdoor gamma dose rate values.

**Figure 5:**
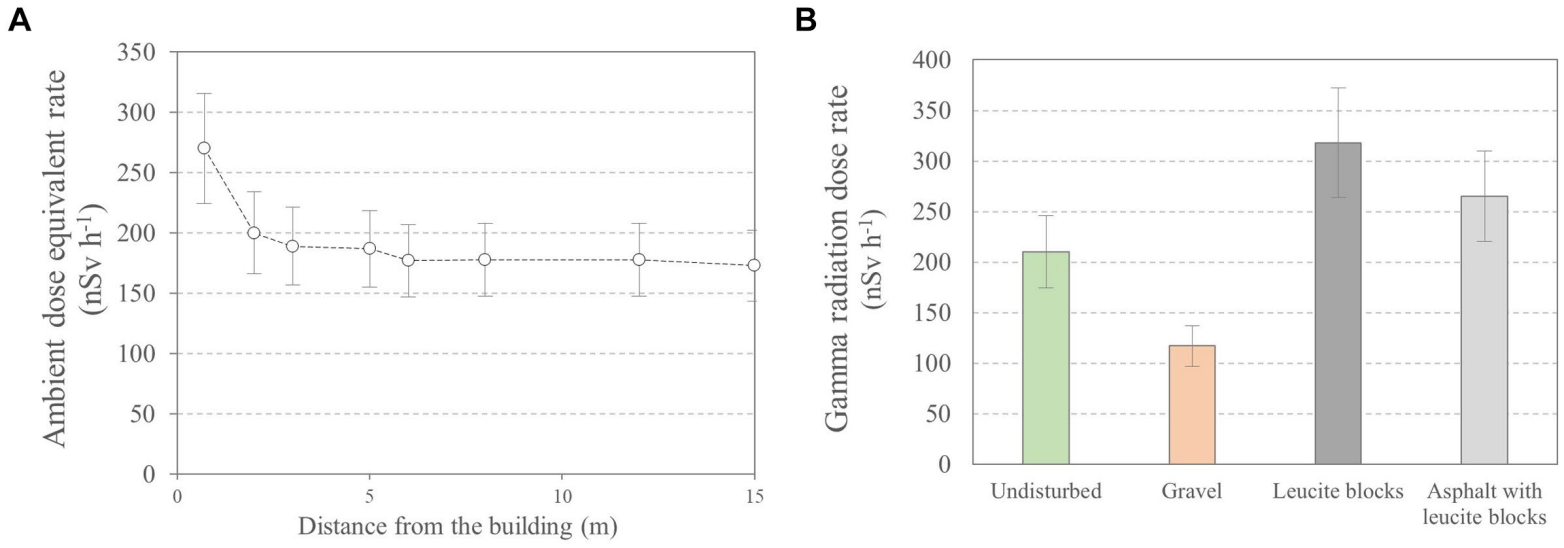
Outdoor gamma ambient dose equivalent rate measured on undisturbed soil at different distances from a building made of Italian tuff **(A)**. The outdoor gamma ambient dose equivalent rate measured at the same distance from the building (2 meters) with different land coverage **(B)**.

The urban structures—land coverage and buildings—affect the outdoor gamma radiation level compared to the undisturbed environments. The land coverage generally acts as a shield to gamma rays produced in the upper soil layer, but both road pavement and buildings may become significant gamma radiation sources when they are made of materials with high NOR activity concentrations, e.g., volcanic materials. The influence of the land coverage typology, as well as the distance from a building constructed of materials with high NOR activity concentrations on the outdoor gamma dose rate, was studied through a small complementary study performed in the garden of the Italian National Institute of Health in Rome. It is a small environment of nearly 5,000 sq. m characterized by different land coverages, i.e., gravel, asphalt, and leucite blocks, and undisturbed areas of lawn, which borders on one side with a building constructed in Italian tuff, an Italian volcanic rock with high NOR activity concentrations. The authors of this study used one of the instruments used for the national campaign to measure the ambient dose equivalent rate. The results are displayed in Figure 5.

An increase in outdoor gamma dose rate value when approaching the building is observed to be up to approximately 15 m due to the gamma rays emitted by the building. The results are consistent with those published by other works [e.g., (10)]. Moreover, the measurements show that different land coverages affect the outdoor gamma dose rate value. The gravel coverage—as an example of a material containing low radionuclide activity concentration—on undisturbed soil reduces the outdoor gamma dose rate value by approximately 40%, while the use of leucite blocks—as an example of a material containing high radionuclide activity concentration—increases the outdoor gamma dose rate value by about 50%. The latter gamma dose rate value is reduced by nearly 20% by adding an asphalt layer on leucite blocks. Similar results were found in the literature for covering materials with low (7, 15) and high NOR activity concentrations (54, 55). This finding leads to high uncertainties when assessing outdoor gamma radiation exposure in urban areas from measurements in undisturbed environments.

**Figure 6:**
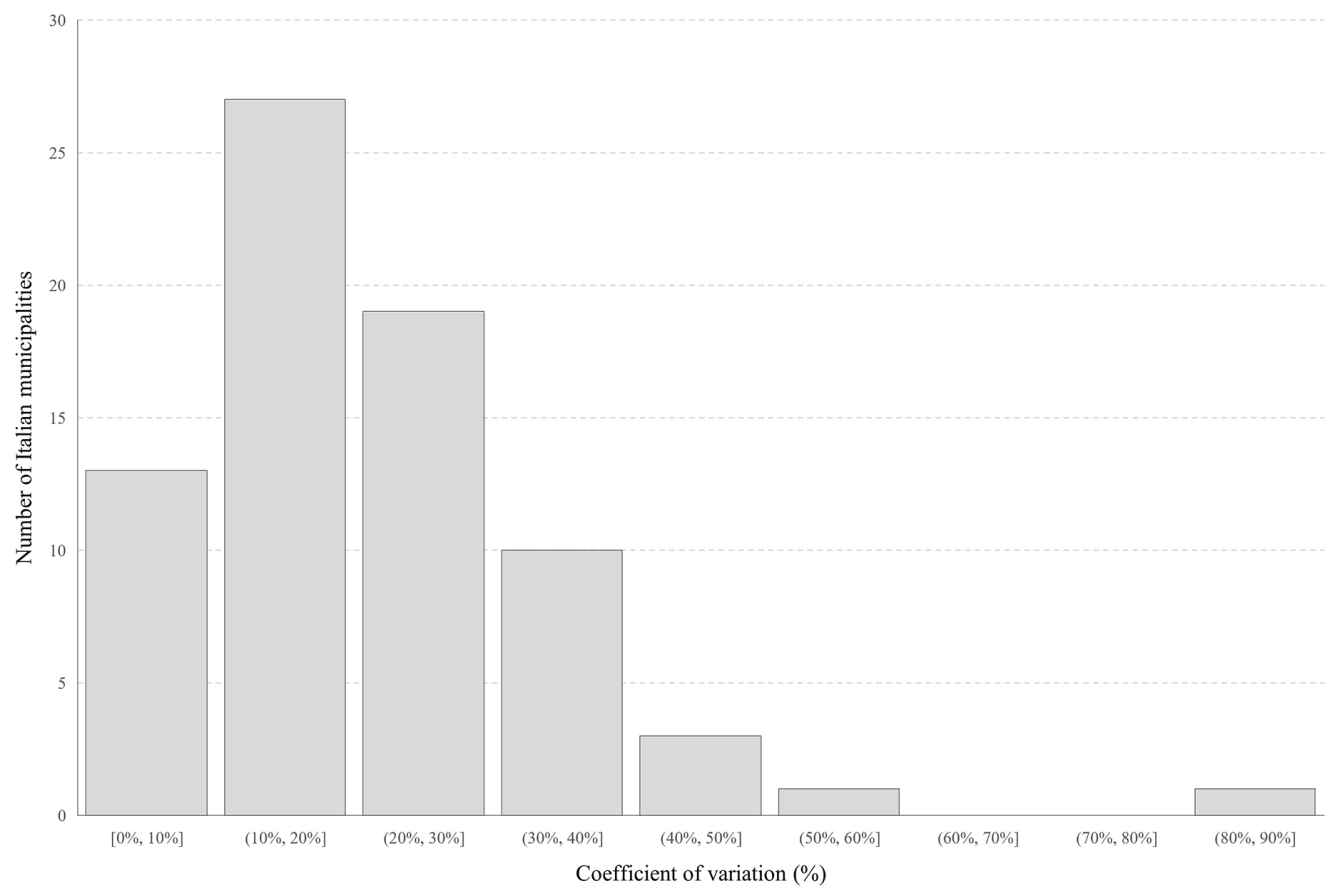
CV distribution for municipalities with more than four measurements.

The outdoor gamma radiation level may strongly vary within the same urban area due to built-up inhomogeneities in terms of materials employed and installation conditions (e.g., thickness and construction typology) and distance of the measurement point from surrounding buildings. The measurement points, i.e., the infrastructures of the telecommunication company, are placed within urban centers in “random” positions relative to land coverage and distance from surrounding buildings (see §2.1), so the computed CV can be useful for estimating the variation of outdoor gamma radiation within urban areas. The average CV was found to be 21%, ranging from 3 to 84% (Figure 6). The first and the third quartiles are 12 and 29%, respectively. These values align with those found in the literature by other authors regarding the spatial variability of outdoor gamma radiation measurements within urban areas (13). The built-up inhomogeneities at the municipal level, the variability of the distance from the surrounding buildings of measurement points, and the strong differences in natural radionuclide content of the soils at the regional level reflect a skewed data distribution for some Italian regions, as shown in Figure 3.

The results of the present study were compared to those of the earlier, and only, Italian national survey on outdoor gamma exposure by Cardinale and Cortellesa (34). These data were critically reviewed by the Italian Institute for Environmental Protection and Research (56).

The comparison concerned only terrestrial gamma dose rate measurements since cosmic radiation is not detected by plastic scintillators, like the instrument adopted in the present study (see §2.3), due to its energy spectrum characterized by high energies (57). The absorbed dose rate values of the 1972 survey were converted into ambient dose equivalent (μSv h^−1^) by adopting 1.2 Sv Gy^−1^ as the conversion factor (40). To compare the results of the two surveys, ambient dose equivalent rate values were expressed in μSv h^−1^ and rounded to two decimal places, being the sensitivity of the instrument used in the 1972 survey 1 μrad h^−1^ (34). The comparison is reported in Table 4.

**Table 4:** Comparison between 1972’s survey outdoor gamma dose rate of terrestrial origin [Cardinale et al. (34)] and outdoor gamma dose rate of the present study.

Italian Region	Ambient dose equivalent rate H*(10) (μSv h^−1^)		Difference (%)
	Cardinale et al. (34)	Present study	
Abruzzo	0.06	0.07	20%
Basilicata	0.11	0.09	−20%
Calabria	0.08	0.14	75%
Campania	0.19	0.19	0%
Emilia-Romagna	0.06	0.09	50%
Friuli Venezia Giulia	0.06	0.07	20%
Lazio	0.16	0.21	30%
Liguria	0.06	0.07	20%
Lombardia	0.07	0.10	45%
Marche	0.07	0.06	−15%
Molise	0.05	0.07	40%
Piemonte	0.07	0.13	85%
Puglia	0.07	0.09	30%
Sardegna	n/a	0.14	n/a
Sicilia	n/a	0.09	n/a
Toscana	0.06	0.08	35%
Trentino-Alto Adige/Südtirol	0.06	0.12	100%
Umbria	0.07	0.07	0%
Valle d’Aosta	0.08	0.17	> 100%
Veneto	0.06	0.09	50%
**Italy**	**0.09**	**0.12**	**30%**

As per the national average, the result of the present survey is 30% higher than that of the 1972 survey. The differences in the regional average gamma dose rate values might be determined by the different sampling strategy of the measuring sites, either in the coverage of the territory or the population. The present study’s measurements have been performed only in urban areas covering all the regions and provinces. In contrast, the measurement points chosen in the 1972 survey were approximately 70% in urban centers and 30% in areas outside the cities, and two regions were not surveyed (Sicilia and Sardegna). Finally, the measurements collected in 1972 were 1,365 in total, which is nearly one-third of those of the present survey. However, no information is available about territory coverage at the provincial or municipal scale.

## 5 Conclusion

A detailed assessment of the Italian population’s exposure to outdoor gamma radiation has been evaluated through 3,876 on-site measurements of the ambient dose equivalent rate within urban areas, where most of the Italian population lives. Among the few reported till date, it is the largest national campaign of on-site outdoor gamma radiation measurements in urban areas carried out worldwide.

The results are representative of the Italian population’s exposure to outdoor gamma radiation in urban areas owing to the high number of measurements and the high population and territory coverage at national and regional levels as well as for most Italian provinces. The measurement points have been chosen at the infrastructures of an Italian telecommunication company distributed in the entire national territory as dense as the residing population. They represent all the possible situations of the population’s outdoor exposure to gamma radiation in urban areas since they were not chosen *a priori* relative to land coverage and distance from surrounding buildings.

The population-weighted national mean of gamma ambient dose equivalent rate resulted in 117 nSv h^−1^, ranging from 62 to 208 nSv h^−1^ at the regional level and from 40 to 227 nSv h^−1^ at the provincial one. Considering these values, the effective dose suffered by the Italian population due to outdoor gamma radiation exposure in urban areas is 0.13 mSv y^−1^, ranging from 0.07 to 0.22 mSv y^−1^ at the regional level.

The spatial variability of outdoor gamma radiation dose rate values increased in urban areas compared to undisturbed environments. In this survey, the spatial variability is 21% on average, ranging from 3 to 74% in terms of CV. The main reason for this variability is the high inhomogeneities of built-up within the cities, strongly affecting the outdoor gamma dose rate. A small complementary study on a limited and controlled area showed (i) an increase in the outdoor gamma radiation dose rate approaching a building constructed of materials containing high NOR activity concentrations, (ii) a decrease in the outdoor gamma radiation dose rate, compared to the undisturbed environment, in case of land coverage with low natural radioactivity content, such as gravel, and (iii) an increase in the outdoor gamma radiation dose rate, compared to the undisturbed environment, in case of land coverage with high natural radioactivity content, such as leucite blocks.

The strategy adopted in this survey, based on a collaboration with a company that owns infrastructures as densely distributed on the national territory as the resident population, was quite feasible, affordable, and time-consuming. These factors allowed us to overcome many difficulties associated with this kind of national representative campaign in terms of logistics and measuring point selection. Therefore, other countries could easily adopt this survey strategy and methodology to conduct surveys on gamma levels in urban environments to better evaluate population exposure.

## Data availability statement

The raw data supporting the conclusions of this article will be made available by the authors, without undue reservation.

## Author contributions

CaC: Conceptualization, Data curation, Formal analysis, Methodology, Visualization, Writing – original draft, Writing – review & editing. AM: Conceptualization, Data curation, Formal analysis, Methodology, Visualization, Writing – original draft, Writing – review & editing. MA: Data curation, Investigation, Writing – review & editing. SA: Formal analysis, Writing – review & editing. MC: Formal analysis, Writing – review & editing. VC: Data curation, Funding acquisition, Resources, Writing – review & editing. CCo: Data curation, Funding acquisition, Resources, Writing – review & editing. ChC: Conceptualization, Visualization, Writing – review & editing. FB: Conceptualization, Funding acquisition, Methodology, Supervision, Writing – review & editing.
